# Zuverlässigkeit von digitalen Gesundheitsinformationen zur Belastungsinkontinenz im Vergleich zwischen verschiedenen Plattformen

**DOI:** 10.1007/s00120-025-02593-7

**Published:** 2025-05-19

**Authors:** Tanja Hüsch, Sita Ober, Anita Thomas, Axel Haferkamp, Matthias Saar, Jennifer Kranz

**Affiliations:** 1 Abteilung für Urologie und Kinderurologie, Universitätsmedizin der Johannes Gutenberg Universität, Langenbeckstr. 1, 55131 Mainz, Deutschland; 2 Praxis Dr. med. Ober & Team, Praxis für Gynäkologie und Geburtshilfe, Michelstadt, Deutschland; 3 Klinik für Urologie und Kinderurologie, Uniklinik RWTH Aachen, Aachen, Deutschland; 4 Klinik für Urologie, Universitätsklinik/Poliklinik und Medizinische Fakultät, Martin-Luther-Universität Halle-Wittenberg, Halle, Deutschland

**Keywords:** Harninkontinenz, Soziale Medien, Digitale Medien, Urogenitale chirurgische Prozeduren, Funktionelle Urologie, Urinary incontinence, Social media, Digital media, Urogenital surgical procedures, Functional urology

## Abstract

**Hintergrund:**

Das Interesse an digitalen Informationen zur Beckenbodeninsuffizienz steigt stetig. Digitale Plattformen bieten hierbei auf einfache und anonyme Weise die Möglichkeit für Betroffene sich über ihre Erkrankung zu informieren. Die Qualität der Informationen innerhalb sowie im Vergleich verschiedener Plattformen ist jedoch nicht hinlänglich bekannt.

**Ziel der Arbeit:**

Das Ziel der vorliegenden Arbeit ist die Untersuchung der Vollständigkeit und Qualität der Informationen zu dem Suchbegriff Belastungsinkontinenz im Vergleich zwischen verschiedenen digitalen Plattformen.

**Material und Methoden:**

Es wurde eine Analyse zur Stichwortsuche „Stress Urinary Incontinence“ auf Google und den sozialen Netzwerken Facebook, YouTube, Instagram und LinkedIn durchgeführt. Es wurden jeweils die ersten 30 Suchergebnisse pro Plattform ausgewertet. Die Ergebnisse wurden nach Informationsgehalt und Lesbarkeit kategorisiert. Zur Beurteilung der medizinischen Qualität wurde das Siegel der Health On the Net (HON) Foundation verwendet.

**Ergebnisse:**

Der Anteil informativer Inhalte war auf YouTube (97 %) und Google (93 %) am höchsten. Inhalte wurden überwiegend von professionellen Organisationen auf Google und YouTube bereitgestellt. Informationen zu konservativen Therapien dominierten in allen Plattformen. Chirurgische Therapieverfahren wurden bei lediglich bis zu 63 % der Ergebnisse in Google und 50 % in YouTube thematisiert. Hierbei lag zudem überwiegend keine vollumfängliche Darstellung aller chirurgischer Optionen vor. Die Lesbarkeit der Texte lag in allen Plattformen nicht laiengerecht vor, auch war eine HON-Zertifizierung nur in Google (37 %) und YouTube (3 %) präsent.

**Schlussfolgerung:**

Die Ergebnisse bieten praktische Einblicke in die Qualität digitaler Informationen zur Belastungsinkontinenz. Sie weisen jedoch Defizite in Lesbarkeit und umfassender Darstellung an chirurgischen Therapien auf. Das Gespräch zwischen therapierender und erkrankter Person bleibt unverzichtbar, um eine vollumfängliche und individuelle Aufklärung der Betroffenen zu gewährleisten.

## Abstract (en)

**Background:**

The interest in digital information on pelvic floor dysfunction is constantly increasing. Various digital platforms offer an easy and anonymous way for patients to seek information about their condition. However, little is known about the quality of the information on the different platforms or about the how the quality of different sites compares.

**Objective:**

The aim of this study was to investigate the completeness and quality of information on the search term “stress urinary incontinence” in comparison between different digital platforms.

**Materials and methods:**

A systematic analysis of the keyword search “stress urinary incontinence” was performed on Google and the social networks Facebook, YouTube, Instagram, and LinkedIn. The first 30 search results on each platform were evaluated. The results were categorized according to information content and readability. The Health On the Net Foundation (HON) seal was used to assess medical quality.

**Results:**

The proportion of informative content was highest on YouTube (97%) and Google (93%). Content was predominantly provided by professional organizations on Google and YouTube. Information on conservative therapies dominated across all platforms. Surgical therapies were only discussed in up to 63% of results on Google and in up to 50% of results on YouTube. In most cases, there was also no comprehensive presentation of all surgical options. The readability of the texts was unsuitable for laypersons on all platforms, and HON certification was only present on Google (37%) and YouTube (3%).

**Conclusion:**

The results offer practical insights into the quality of digital information on stress urinary incontinence. However, they show deficits in readability and comprehensive presentation of surgical therapies. The physician–patient relationship remains indispensable for taking individual needs into account and avoiding misinformation.

## Hinführung zum Thema

Patientinnen informieren sich zunehmend anhand von digitalen Plattformen über ihre Erkrankung, bevor sie das Gespräch zwischen therapierender und erkrankter Person führen. Dennoch liegen keine Informationen über die Vollständigkeit und Qualität der Informationen im Vergleich zwischen den verschiedenen digitalen Plattformen vor. Schwer verständliche Texte oder unvollständige Informationen können prinzipiell zur Fehlinformation und Schädigung des Verhältnisses zwischen therapierender und erkrankter Person führen.

In der vorliegenden Arbeit wurde daher der Suchbegriff Belastungsinkontinenz im Vergleich zwischen verschiedenen digitalen Plattformen hinsichtlich der Vollständigkeit sowie Qualität der thematischen Informationen untersucht.

## Hintergrund und Fragestellung

Die Digitalisierung gewinnt in unserer Gesellschaft zunehmend an Bedeutung [1, 2]. Die Medizin ist auf vielfältige Weise von diesem Wandel betroffen. Zunehmend informieren sich Betroffene vor Erstvorstellung bei ihrer behandelnden Person online über ihr Erkrankungsbild und sind damit bereits häufig vorinformiert. Dieser Trend kann die Beziehung zwischen therapierender und erkrankter Person auf zwei unterschiedliche Arten beeinflussen: Einerseits kann das bereits vorab erworbene Wissen den weiteren Diagnostik- bzw. Therapiepfad vereinfachen und optimieren [3], andererseits besteht die Gefahr, dass Fehlinformationen die Beziehung zwischen behandelnder und erkrankter Person belasten und sich negativ auf den Behandlungsverlauf auswirken [4].

Das Interesse an digitalen Informationen zur Beckenbodeninsuffizienz und damit verbundenen Krankheitsbildern wächst stetig [5]. Die hohe Prävalenz dieser gutartigen Krankheitsbilder spielt hierbei womöglich eine wesentliche Rolle. Schätzungen zufolge liegt eine Harninkontinenz bei 25–45 % aller Frauen vor, während sie bei Männern etwa die Hälfte dieses Wertes beträgt [5]. Ebenso wird eine hohe globale Prävalenz für das Risiko eines Beckenbodenprolapses berichtet, welche bis ca. 51 % betragen kann, wobei v. a. Frauen im Alter von über 50 Jahren betroffen sind [6].

Betroffenen stehen einer Vielzahl von digitalen Plattformen zur Verfügung, um sich anonym und niederschwellig über ihre Erkrankung zu informieren [7, 8]. Google gilt derzeit als die global beliebteste Suchmaschine mit mehr als 8,5 Mrd. Suchanfragen pro Tag, welche sie hiermit auch zu der am häufigsten besuchen Website macht [9, 10]. Die derzeit beliebtesten sozialen Medien umfassen Instagram, Facebook, LinkedIn und YouTube. LinkedIn ist ein soziales Netzwerk, das sich auf geschäftliche und berufliche Beziehungen konzentriert und eine bedeutende Anzahl medizinischer Fachkräfte umfasst. Aktuell zählt es etwa 756 Mio. registrierte Mitglieder aus 200 Ländern. Die Plattform ermöglicht das Veröffentlichen von Beiträgen und das Teilen von Artikeln mit dem eigenen Netzwerk [11]. Instagram ist eine Plattform, die sozialen Austausch durch das Teilen von Fotos, Videos und anderen bewegten Bildern ermöglicht. Mit über 1 Mrd. registrierter Nutzenden und > 500 Mio. täglich aktiven Nutzenden zählt sie zu den weltweit größten sozialen Netzwerken. [12, 13]. YouTube ist eine Online-Plattform, die als Videoportal zum Teilen von Videos dient, wobei diese auch kommentiert werden können [14]. Täglich werden auf YouTube rund 1 Mrd. h Videomaterial angesehen [15], womit sie die größte Videoplattform weltweit repräsentiert [16]. Mit über 2,49 Mrd. monatlich aktiven Nutzenden ist YouTube nach Google die am zweithäufigsten besuchte Website weltweit und nach Facebook das am häufigsten genutzte soziale Netzwerk [14]. Facebook ist ein soziales Netzwerk mit über 2,8 Mrd. monatlich aktiven Nutzenden. Die Plattform ermöglicht es, Texte, Fotos, Videos und andere Inhalte zu teilen sowie direkt mit anderen Nutzenden zu interagieren [17].

In der vorliegenden Arbeit wurde die Vollständigkeit sowie Qualität der Informationen zu dem Suchbegriff Belastungsinkontinenz hinsichtlich Pathophysiologie, Diagnostik und Therapie in digitalen Plattformen untersucht. Hierzu wurden die derzeit beliebtesten sozialen Netzwerke sowie die Suchmaschine Google herangezogen. Das Ziel der Arbeit war eine Bewertung der einzelnen Plattformen zur objektiven Beurteilung des Inhalts der herangezogenen Informationen für Betroffene zum Thema Belastungsinkontinenz.

## Studiendesign und Untersuchungsmethoden

Zwischen Januar und Juni 2021 wurde eine systematische Analyse der Stichwortsuche „Stress Urinary Incontinence“ in Google und den sozialen Netzwerken Facebook, YouTube, Instagram und LinkedIn durchgeführt. Recherchen wurden im Inkognitomodus bzw. mit geschlechtsneutralen Accounts durchgeführt, um personalisierte Ergebnisse zu vermeiden. Pro Plattform wurden die ersten 30 Suchergebnisse ausgewertet. Grundlage hierfür war die Auswertung von drei sog. „search engine results pages“ (SERP), was jeweils 10 Ergebnissen pro Seite auf Google entspricht. Studien belegen, dass < 6 % der Nutzenden Ergebnisse betrachten, die über die dritte SERP hinausgehen. Zudem ist nachgewiesen, dass 70 % der Nutzenden ausschließlich die ersten 10 Suchergebnisse beachten [18, 19], sodass der Einschluss von 30 Sucherergebnissen als relevant für eine objektive Beurteilung der verwendeten Suchergebnissen von Nutzenden angesehen werden kann.

Die Suchergebnisse wurden nach Informationsgehalt, Werbung und persönlichen Erfahrungen kategorisiert. Informative Inhalte umfassten Angaben zu Pathophysiologie, Diagnostik und Therapie. Irrelevante Ergebnisse wie Jobangebote wurden als nicht informativ bewertet. Informative Beiträge wurden zudem hinsichtlich Autorenschaft (Ärzte, Betroffene, Organisationen, Industrie) und assoziierter Krankheitsbilder analysiert.

Für Google-Ergebnisse erfolgte eine zusätzliche Bewertung der Lesbarkeit und Verständlichkeit mittels der Flesch-Kincaid-Readability-Skala [20]. Diese bewertet Texte basierend auf Satz- und Wortlängen und richtet sich nach dem U.S.-amerikanischen Schulsystem. Ein Wert von 8 wird als allgemein verständlich angesehen, während Patienteninformationen idealerweise einen Wert von 5–6 erreichen sollten.

Zur Bewertung der medizinischen Qualität wurde das Sofortanalyse-Tool der Health On the Net (HON) Foundation eingesetzt, welches über ein Browser-Siegel eine unmittelbare medizinische Zertifizierung von Webseiten anzeigt. Die Stiftung ist eine gemeinnützige Nichtregierungsorganisation, die sich der Bewertung der Qualität medizinischer Informationen auf Webseiten widmet und diese, bei entsprechender Güte, entsprechend kennzeichnet [21].

Es wurde eine deskriptive Statistik durchgeführt. Univariate Analysen wurden mit dem χ^2^-Test für kategoriale Variablen und der unabhängige t‑Test für kontinuierliche Variablen zur Evaluierung von Unterschieden zwischen den Gruppen verwendet. Das Signifikanzniveau wurde auf < 5 % festgelegt.

Die statistische Auswertung wurde mit IBM® SPSS® (IBM, Armonk, NY, USA) Version 26 durchgeführt. Graphische Darstellungen erfolgten mittels Microsoft Office Excel®(Microsoft Corporation, Redmond, WA, USA).

## Ergebnisse

**Abb. 1:**
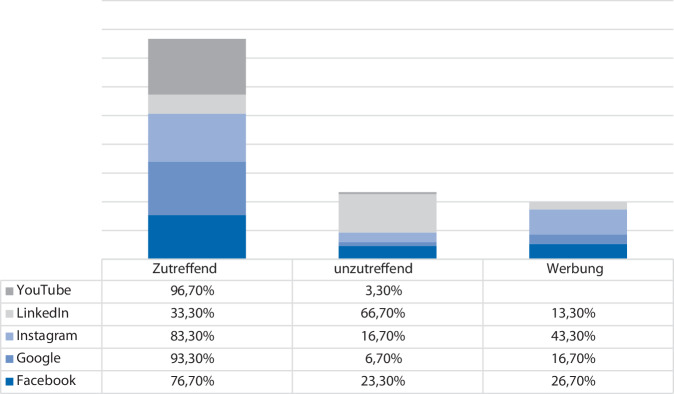
Prozentualer Anteil zutreffender Informationen sowie Werbung zum Suchbegriff Belastungsinkontinenz aufgeteilt nach Plattform

Der Anteil von informativen Inhalten war auf den Plattformen YouTube und Google mit jeweils 97 % und 93 % am höchsten. Unzutreffende Inhalte waren mit 67 % v. a. auf LinkedIn vorhanden, wobei der höchste Werbeanteil mit 43 % bei Instagram vorlag (Abb. 1). Inhalte von persönlichen Erfahrungen waren überwiegend auf YouTube (23 %) und Facebook (20 %) vorliegend. Der Anteil an Inhalten von Betroffenen lag bei 13 % in Facebook, 10 % auf Instagram und 3 % auf YouTube. Google und LinkedIn beinhalteten keine Ergebnisse von Betroffenen. Die Inhalte wurden überwiegend von professionellen Organisationen auf Google und YouTube veröffentlich. Medizinisches Fachpersonal veröffentlichte überwiegend Inhalte auf Instagram (70 %), YouTube (67 %) und LinkedIn (57 %), wobei PhysiotherapeutInnen die hierbei größte Berufsgruppe darstellte. UrologInnen veröffentlichten Inhalte am häufigsten auf LinkedIn (23 %) gefolgt von YouTube (20 %). Industriegesponserte Inhalte waren mit jeweils 27 % auf LinkedIn und Facebook am höchsten.

Zu dem Suchbegriff wurden assoziierte Krankheitsbilder beschrieben, welche die überaktive Harnblase, Mischinkontinenz, Beckenbodeninsuffizienz, Stuhlinkontinenz, sexuelle Einschränkungen, Geburtsverletzungen, Schlafstörungen und emotionalen Stress umfassen. Die am häufigsten assoziierten Krankheitsbilder waren Schlafstörungen und emotionaler Stress (Tab. 1). Die Diagnostik der Belastungsinkontinenz wurde in 67 % und 40 % der Suchergebnisse auf Google und YouTube thematisiert. In Facebook und Instagram wurde die Diagnostik in jeweils 10 % und 3 % thematisch behandelt, wobei keine Informationen in LinkedIn vorlagen.

**Tab. 1:** Krankheitsbilder, die in Bezug auf den Suchbegriff Belastungsinkontinenz genannt wurden, aufgeführt nach Vorkommen auf jeweiliger digitaler Plattform

	Facebook (%)	Google (%)	Instagram (%)	LinkedIn (%)	YouTube (%)	*p*-Wert
Unspezifisch	43	60	97	17	33	< 0,001^a^
Überaktive Blase	13	43	37	3	30	0,006^a^
Belastungsharninkontinenz	63	83	90	30	90	< 0,001^a^
Mischinkontinenz	17	43	30	3	27	0,002^a^
Beckenbodeninsuffizienz	17	20	33	7	10	0,002^a^
Stuhlinkontinenz	3	10	0	0	0	0,179
Sexuelle Einschränkung	10	30	37	10	0	0,402
Geburtsverletzungen	20	37	27	7	40	0,031
Schlafstörungen/Emotionaler Stress	43	70	43	7	60	< 0,001^a^

^a^*p-*Wert signifikant < 0,05

**Abb. 2:**
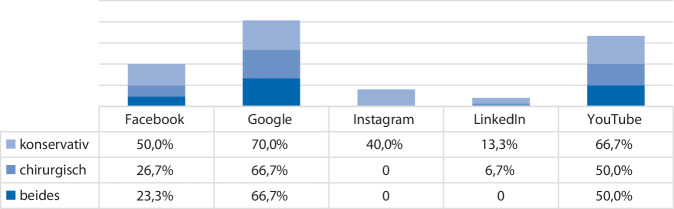
Prozentualer Anteil an Informationen zu konservativen vs. chirurgischen Therapieformen aufgeteilt nach Plattform zum Suchbegriff Belastungsinkontinenz

In Hinblick auf therapeutische Informationen überwogen in allen Plattformen Inhalte zu konservativen Maßnahmen. Der Anteil an Informationen zu chirurgischen Verfahren lag nur in maximal 67 % der Informationen auf Google sowie in der Hälfte der Fälle bei YouTube vor. Die Informationen zu chirurgischen Verfahren fehlten gänzlich auf Instagram (Abb. 2).

Bei den konservativen Therapieoptionen wurde Beckenbodengymnastik als die am häufigsten empfohlene Maßnahme identifiziert, wobei v. a. praktische Anleitungen auf Instagram vermittelt wurden. Pharmakotherapie und neuromodulatorische Verfahren wurden in der Hälfte der Suchergebnisse auf Google erwähnt (Tab. 2).

**Tab. 2:** Prozentualer Anteil an Informationen zu spezifischen Therapieoptionen aufgeteilt nach Plattform zum Suchbegriff Belastungsinkontinenz

	Facebook (*n* [%])	Google (*n* [%])	Instagram (*n* [%])	LinkedIn (*n* [%])	YouTube (*n* [%])
*Konservativ*					
Pharmakotherapie	2 (6,7)	15 (50)	0	2 (6,7)	6 (20)
Beckenbodengymnastik	13 (43,3)	21 (70)	11 (36,7)	2 (6,7)	6 (20)
Biofeedback	2 (6,7)	9 (30)	0	0	0
Akupunktur, Neuromodulation (PTNS etc.)	7 (23,3)	15 (50)	1 (3,3)	1 (3,3)	11 (36,7)
Alternative Therapien (Homöopathie etc.)	0	0	0	1 (3,3)	0
*Chirurgisch*					
Midurethrale Schlingen	7 (23,3)	19 (63,3)	0	2 (6,7)	19 (63,3)
Minischlingen	1 (3,3)	17 (56,7)	0	2 (6,7)	7 (23,3)
„Bulking agents“	1 (3,3)	17 (56,7)	0	0	8 (26,7)
Autologe Faszienschlinge	0	0	0	0	1 (3,3)
Kolposuspension nach Burch	2 (6,7)	16 (53,3)	0	0	8 (26,7)

Unter den chirurgischen Therapieoptionen wurde die midurethrale Schlinge in 63 % der Suchergebnisse auf Google und YouTube zum Suchbegriff „Belastungsinkontinenz“ aufgeführt. Andere Therapieansätze, wie die autologe Faszienschlinge oder alternative Methoden, wurden entweder gar nicht oder nur in etwa der Hälfte der chirurgischen Suchergebnisse erwähnt (Tab. 2).

**Abb. 3:**
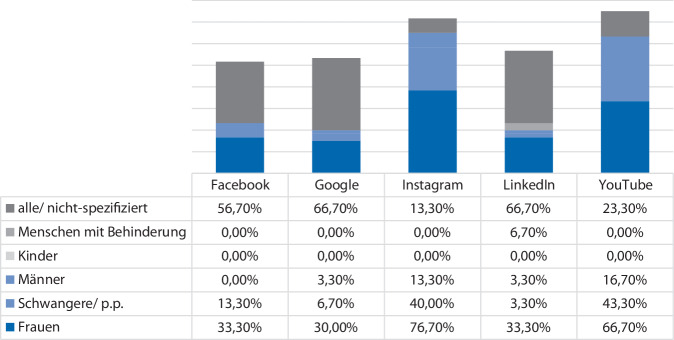
Prozentuale Aufteilung der Information nach Zielgruppe zum Suchbegriff Belastungsinkontinenz (Mehrfachnennungen waren möglich)

Die meisten Informationen waren entweder zielgruppenunspezifisch oder an Frauen gerichtet. Kinder wurden auf keiner der Plattformen als Zielgruppe erwähnt, während Menschen mit Behinderung lediglich auf LinkedIn in 6,7 % der Fälle als Zielgruppe angesprochen wurden. Männer waren auf allen Plattformen mit einem Anteil von < 20 % ebenfalls unterrepräsentiert (Abb. 3).

Eine HON-Zertifizierung lag lediglich bei Google und YouTube in jeweils 37 % und 3 % der Suchergebnisse vor. Der Lesbarkeitsindex lag bei Google bei 9, bei Facebook und Instagram 8 und bei LinkedIn bei 7.

## Diskussion

In der vorliegenden Arbeit wurde der quantitative Informationsgehalt und dessen Güte zu dem Suchbegriff Belastungsinkontinenz auf den derzeit am häufigsten verwendeten sozialen Netzwerken sowie der Suchmaschine Google untersucht. Mit der hiesigen plattformübergreifenden Untersuchung ist die vorliegende Arbeit einzigartig, da derzeit vorliegende Untersuchungen sich vornehmlich auf eine Plattform beschränken und somit keine vergleichenden Ergebnisse vorliegen [18, 19].

Die digitalen Plattformen mit dem höchsten Anteil an informativen Inhalten, erstellt durch überwiegend professionelle Organisationen, innerhalb der ersten 30 Suchergebnisse waren Google und YouTube. Überraschenderweise war der Anteil an persönlichen Erfahrungen durch Betroffene mit maximal 23 % der Sucherergebnisse gering. Eine mögliche Erklärung hierfür kann die Angst vor möglichen Online-Shamings bei Offenlegung dieses schambehafteten Themas sein. Betroffene können durch die Niederschwelligkeit und Anonymität im digitalen Raum rasch einer Ausgrenzung oder unkontrollierbaren Bloßstellung ausgesetzt sein, welche fatale Folgen für die Betroffenen nach sich ziehen kann. Obwohl auch positive Effekte durch die Präsentation im öffentlichen Raum möglich sind, überwiegt die Furcht vor Online-Shaming und deren Konsequenzen [22].

Die Plattform mit dem geringsten Anteil an informativen Inhalten stellte LinkedIn dar, wobei andere Nachrichten wie beispielsweise Jobangebote oder Werbung dominierten. Dieses Ergebnis stimmt mit dem anderer Studien überein [23, 24]. Zusammenfassend war es überraschend, dass der Anteil an informativen Inhalten auf LinkedIn überwiegend nicht relevant war, sodass diese Plattform als Informationsquelle als vernachlässigbar bei der Thematik Belastungsinkontinenz einzustufen ist.

Der Lesbarkeitsindex lag bei den Plattformen zwischen 7–9, womit keine den empfohlenen Mindestindex von 6 für eine laiengerechte Sprache erreicht hatte. Der Zugang für Betroffene wird hierdurch eingeschränkt und birgt die Gefahr der Fehlinformation durch falsche Interpretation der Inhalte. Die fehlende laiengerechte Veröffentlichung von Informationen ist hinlänglich bekannt und konnte durch die vorliegende Arbeit erneut bestätigt werden [25, 26].

Der informative Anteil war bei YouTube am größten und lag hiermit sogar über Google. Eine mögliche Erklärung könnte der Algorithmus von Suchmaschinen zur Videosortierung geben. Es besteht die Vermutung, dass die Anzahl der Nutzer einen Hinweis auf die Qualität liefern könnte [27]. Aufgrund der fehlenden Offenlegung dieser Information ist eine abschließende Erklärung nicht möglich. Aktuelle Entwicklungen deuten darauf hin, dass digitale Informationen künftig verstärkt in einer laiengerechten Sprache aufbereitet werden. So führte YouTube in Deutschland Ende Februar 2023 ein eigenes Gütesiegel namens „YouTube Health“ für Gesundheitsinformationen ein [28].

Themenspezifisch lagen Informationen zur Pathophysiologie und Diagnostik überwiegend auf YouTube und Google vor. Der Anteil an konservativen Therapieoptionen dominierte in allen Plattformen. Soziale Medien wie Instagram fokussierten sich dabei überwiegend auf ein spezifischen Themengebiet der Beckenbodengymnastik, welche in Übungen vorgeführt wurden. Interessanterweise waren die chirurgischen Therapieverfahren unterrepräsentiert. Es konnte nachgewiesen werden, dass nur in knapp der Hälfte der Suchergebnisse auf YouTube und Google chirurgische Therapieverfahren überhaupt genannt wurden. Sofern eine Nennung erfolgte, waren oftmals keine vollumfängliche Information über alle zur Verfügung stehenden Verfahren vorliegend. Die am häufigsten genannte Therapieoption war hierbei die midurethrale Schlinge. Zusammenfassend war der Informationsgehalt zu chirurgischen Therapien der Belastungsinkontinenz auf allen Plattformen unzureichend. Dieses Ergebnis konnte bereits in anderen Untersuchungen zum Themengebiet der funktionellen Urologie nachgewiesen werden [23].

Eine HON-Zertifizierung lag lediglich bei Google und YouTube vor, wobei der Anteil bei jeweils 37 % und 3 % lag. Obwohl die Anzahl der Zertifizierungen gering ist, deckt sich die Anzahl in der hiesigen Untersuchung mit anderen. Weiterhin zeigt sich ein genereller Trend für eine Zunahme an Zertifizierungen [29, 30].

Als Limitierung dieser Arbeit ist die Anzahl der untersuchten Ergebnisse pro Plattform zu nennen. Dennoch ist hierbei zu berücksichtigen, dass die überwiegende Anzahl an Nutzenden nicht mehr als 10 Ergebnisse aufrufen. Somit ist die Beschränkung auf 30 Suchergebnisse relevant und spiegelt eine realistische Darstellung der genutzten Informationen dar. Weiterhin ist das Ergebnis durch die Dynamik der digitalen verfügbaren Informationen und Aktualisierungen möglicherweise Schwankungen unterworfen. Dennoch konnte in der hiesigen Arbeit ein prinzipieller Trend der vorhandenen Informationen und Informationslücken identifiziert werden, welche erwartungsgemäß nicht kurz- bzw. mittelfristig größeren Änderungen unterworfen sind.

## Schlussfolgerung

Die vorliegenden Ergebnisse geben eine praktische Übersicht zur Vollständigkeit und Qualität der Informationen zur Belastungsinkontinenz auf verschiedenen digitalen Plattformen. Diese können zur Bewertung und Unterstützung von Betroffenen praktisch eingesetzt werden. Dennoch sollte auf die eingeschränkte Lesbarkeit sowie fehlende vollumfängliche Darstellung v. a. zu möglichen Therapieoptionen der Belastungsinkontinenz hingewiesen werden. Es wird hiermit ebenso bestätigt, dass das Gespräch zwischen therapierender und erkrankter Person weiterhin unerlässlich in der klinischen Praxis ist, um eine ganzheitliche Beratung zu gewährleisten und auf individuelle Bedürfnisse der Betroffenen eingehen zu können.

Obwohl die Anzahl an zertifizierten Informationen stetig zunimmt, sollten auch Fachgesellschaften kritisch ihre Verantwortung der zur Verfügung gestellten laiengerechter Informationen zu verschiedenen Krankheitsbildern überdenken.

## Fazit für die Praxis

Kernaussagen und konkrete Handlungsanweisungen:

- Die Vollständigkeit und Qualität der Informationen zur Belastungsinkontinenz variieren stark zwischen den verschiedenen digitalen Plattformen.
- YouTube und Google weisen den höchsten Anteil an informativen Inhalten zu der Thematik auf.
- LinkedIn hat überwiegend keine relevanten Informationen zur Pathophysiologie, Diagnostik oder Therapie.
- Inhalte zu konservativen Therapieoptionen dominiert alle Plattformen.
- Informationen zu chirurgischen Therapiemaßnahmen sind in maximal 64 % der Suchergebnisse auf Google zu finden, wobei jedoch auch keine vollständige Darstellung aller Verfahren gewährleistet ist.
- Die vorliegenden Ergebnisse geben praktische Hilfestellungen zur Beratung von Betroffenen, welche digitalen Plattformen bei Bedarf zum Thema Belastungsinkontinenz bevorzugt verwendet werden können.
- Das ärztliche Gespräch bleibt weiterhin ein unersetzlicher Baustein in der Betreuung von Betroffenen.

## Data Availability

Die erhobenen Datensätze können auf begründete Anfrage in anonymisierter Form beim korrespondierenden Autor angefordert werden.
